# A new approach to produce [^18^F]MC225 via one-step synthesis, a PET radiotracer for measuring P-gp function

**DOI:** 10.1186/s41181-021-00139-8

**Published:** 2021-07-15

**Authors:** Lara Garcia-Varela, Khaled Attia, John Carlo Sembrano, Olivier Jacquet, Inês F. Antunes, Chantal Kwizera, Ton J. Visser, Rudi A. J. O. Dierckx, Philip H. Elsinga, Gert Luurtsema

**Affiliations:** 1 Department of Nuclear Medicine and Molecular Imaging, University of Groningen, University Medical Centre Groningen, Hanzeplein 1, 9713 GZ Groningen, the Netherlands; 2 Symeres, Kadijk 3, 9747 AT Groningen, the Netherlands

## Abstract

**Background:**

[^18^F]MC225 is a radiotracer for imaging P-glycoprotein (P-gp) function at the blood-brain barrier. The P-gp function can be altered due to different factors, for instance, decreased P-gp function has been described in patients with Alzheimer’s or Parkinson’s Disease. The current applied radiosynthesis of [^18^F]MC225 involves 2 steps, including the distillation of the [^18^F] fluoroethylbromide intermediate. To develop a more robust synthetic procedure, it is of interest to produce the radiotracer via a 1-step synthesis. The present study describes a new synthetic approach to produce [^18^F]MC225 via direct ^18^F-fluorination. Moreover, we also provide the appropriate conditions for the automation of the synthesis. A mesylate precursor was synthesized via a multi-step synthetic route and used for the radiolabeling. The nucleophilic substitution of the mesylate group by [^18^F] Fluoride was automated in two different synthesis modules: IBA Synthera and Eckert and Ziegler PharmTracer (E&Z).

**Results:**

The mesylate precursor was synthesized in 7 steps starting with 5-hydroxy-1-tetralone (commercially available) in practical yields. The stability of the precursor was improved via mesylate salt formation method. The radiolabeling was done by adding the mesylate precursor dissolved in DMF to the dried [^18^F]KF/K_2.2.2_ complex and heating at 140 °C for 30 min. Quality control by UPLC confirmed the production of [^18^F]MC225 with a molar activity (A_m_) higher than 100 GBq/micromole. The synthesis time in Synthera was 106 min and the product was obtained with a radiochemical purity higher than 95% and RCY of 6.5%, while the production in E&Z lasted 120 min and the product had a lower radiochemical purity (91%) and RCY (3.8%).

**Conclusions:**

[^18^F]MC225 was successfully produced via a 1-step reaction. The procedure is suitable for automation using commercially available synthesis modules. The automation of the radiosynthesis in the Synthera module allows the production of the [^18^F]MC225 by a reliable and simple method.

## Background

[^18^F]MC225 is a radiotracer for imaging P-glycoprotein (P-gp) function at the blood-brain barrier (BBB) (Savolainen et al., 2015; Savolainen et al., 2017). P-gp is an efflux transporter located in the luminal side of the cerebral endothelial cells which constitute the main component of the BBB (Appelboom et al., 2016; Abbott et al., 2010). P-gp belongs to the ATP-Binding Cassette transporter family, their function is ATP-dependent and apart from the BBB, these transporters are also located in several tissues involved in absorption and excretion functions such as the intestine, testes, placenta, kidneys, and liver (Mahringer & Fricker, 2016). In the BBB, the main function of P-gp is to transport a wide variety of substances out of the brain to the blood. Therefore, this transporter contributes to limit the permeability of the BBB and protects the Central Nervous System (CNS) from neurotoxic compounds (Mahringer & Fricker, 2016).

However, the P-gp function can be altered due to different factors. Many unrelated xenobiotic compounds can increase the P-gp expression and function which leads to reduced concentration of drugs in the desired target causing decreases in drug efficacy (Wen et al., 2019; Durk et al., 2015; Brenn et al., 2014; Perloff et al., 2004; Bauer et al., 2004). Moreover, dysfunctions in P-gp function have been observed in various disease conditions (Mahringer & Fricker, 2016). For instance, patients with intractable epilepsy, have shown increased P-gp expression and function in isolated brain capillaries (Feldmann & Koepp, 2012; Hartz et al., 2017). Also, a decreased P-gp function has been reported in Alzheimer’s and Parkinson’s disease (Löscher & Potschka, 2005). Therefore, the assessment of P-gp function using Positron Emission Tomography (PET) imaging can help to improve the diagnosis of certain CNS diseases where the P-gp function is altered. In addition, this technique can also evaluate treatments that affect P-gp function (Kannan et al., 2009).

[^18^F]MC225 has been developed as a weak substrate of the P-gp transporter (Savolainen et al., 2015). Thus, this radiotracer showed a higher brain uptake at baseline conditions (Savolainen et al., 2017), which allows us to measure both increased and decreased P-gp function at the BBB in rats (Savolainen et al., 2017). Previous evaluation of the radiotracer in vivo has shown good pharmacokinetic properties, an adequate signal-to-noise ratio, high sensitivity toward the target, and low levels of radio-metabolites inside the brain. All these characteristics make [^18^F]MC225 a suitable radiotracer (Savolainen et al., 2017; García-Varela et al., 2020) and worthwhile to perform clinical studies to evaluate the P-gp function in the human brain.

**Fig. 1:**
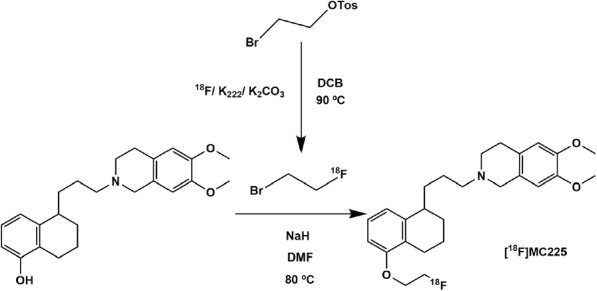
the 2-step synthesis of [^18^F]MC225

For the validation of [^18^F]MC225 for clinical use, the production must be compliant with Good Manufacturing Practice (GMP). The currently used GMP-synthesis of [^18^F]MC225 (Fig. 1) implies two synthesis steps, including a distillation step of the [^18^F] fluoroethylbromide intermediate. Unfortunately, this relatively time-consuming and complex procedure results in low radiochemical yields (Savolainen et al., 2015). Thus, it may be convenient to simplify the radiotracer synthesis via 1-step procedure to make the reaction simpler, and thus more robust.

Therefore, this study aims to produce [^18^F]MC225 via a 1-step reaction using a new MC225 precursor compound which allows the production of the radiotracer by direct ^18^F-fluorination. Moreover, the study discusses the most appropriate conditions for the automation of the synthesis.

## Methods

### Synthesis of phenol precursor (Fig. 2)

**Fig. 2:**
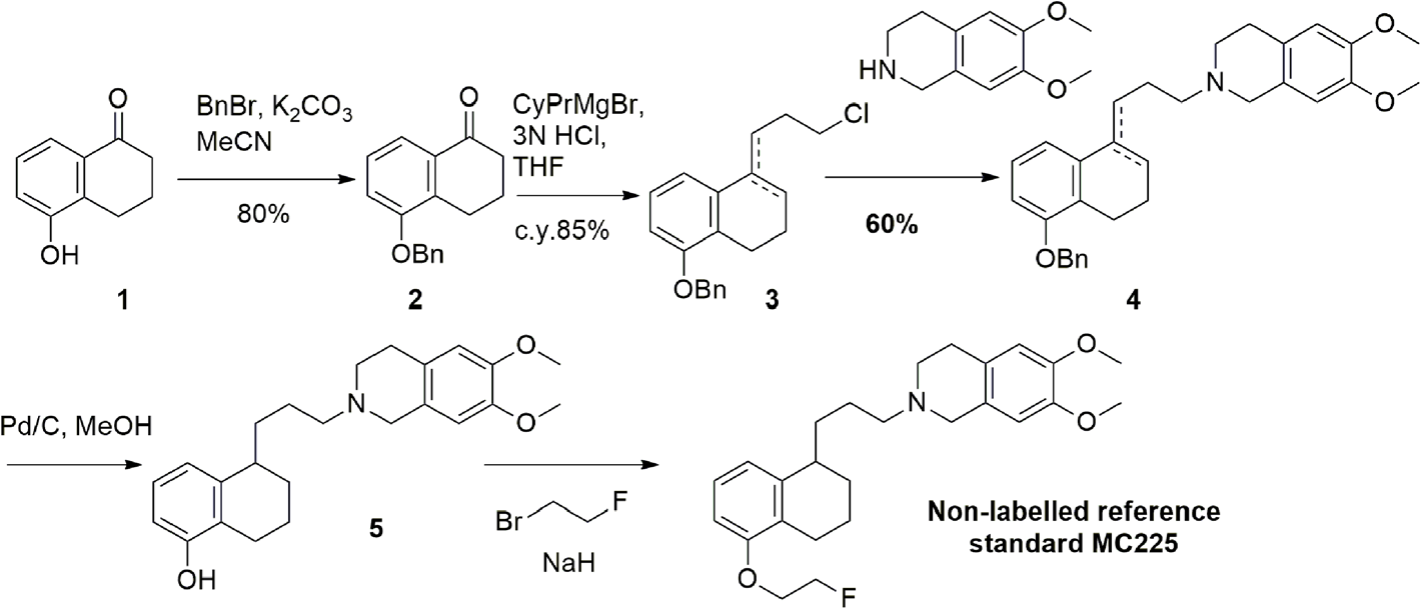
Alternative route for synthesizing the phenol precursor

5-[3-(6,7-Dimethoxy-3,4-dihydro-1H-isoquinolin-2-yl)-propyl]-5,6,7,8-tetrahydro-naphthalen-1-ol, called the phenol precursor was synthesized as previously described (van Waarde et al., 2009) but with some modifications on the synthesis route (Fig. 2).

Step 1: 5-(benzyloxy)-3,4-dihydronaphthalen-1(2H)-one (2).

5-Hydroxy-1-tetralone is commercially available (Combi Blocks) and was protected with a benzyl group as follow: 5-Hydroxy tetralone (1, 5.0 g, 30.9 mmol, 1.0 eq.) and potassium carbonate (8.5 g, 61.7 mmol, 2.0 eq.) dissolved in ACN were stirred for 15 min at room temperature under N_2_-atmosphere. Benzyl bromide (4.04 mL, 33.9 mmol, 1.1 eq.) was added and the resulting beige suspension was stirred overnight at room temperature under N_2_-atmosphere. The reaction mixture was concentrated in vacuum and the crude material was partitioned between water (50 mL) and DCM (50 mL). The layers were separated, and the organic layer was successively washed with water (50 mL), a saturated aqueous solution of NaHCO_3_ (50 mL), and brine (50 mL). The organic layer was collected, dried with anhydrous Na_2_SO_4_, filtered, and concentrated in vacuum to give a beige oil which slowly solidified on standing. The solids were triturated with heptane (30 mL) to give 5-(benzyloxy)-3,4-dihydronaphthalen-1(2H)-one (2) as a crystalline white solid (6.5 g, 83% of yield). ^1^H-NMR (300 MHz, CDCl_3_) δ 7.68 (dd, J = 7.9, 1.1 Hz, 1H), 7.54–7.18 (m, 7H), 7.08 (dd, J = 8.1, 1.2 Hz, 1H), 5.11 (s, 2H), 2.98 (t, J = 6.2 Hz, 2H), 2.64 (dd, J = 7.5, 5.7 Hz, 2H), 2.27–1.97 (m, 2H).

Step 2: Mixture of 8-(benzyloxy)-4-(3-chloropropyl)-1,2-dihydronaphthalene and (*E*)- 5-(benzyloxy)-1-(3-chloropropylidene)-1,2,3,4-tetrahydronaphthalene (3).

For the next step, the benzylated product (compound 2) was reacted with freshly prepared Grignard reagent. Magnesium turnings (0.96 g, 39.7 mmol, 2.0 eq.), anhydrous THF (30 mL), and iodine (1 grain, catalytic) were combined in a flame-dried three-necked flask under N_2_-atmosphere. Cyclopropyl bromide (2.4 mL, 39.7 mmol, 2.0 eq.) was added portion-wise (total addition time = 15 min). After the complete addition of the bromide, the mixture was slowly heated to 55 °C. The color of the mixture turned from brown to colorless accompanied by slight effervescence. The resulting mixture was stirred at 55 °C for 2 h at room temperature under N_2_-atmosphere. At this point, the color of the reaction turned yellowish and the magnesium turnings were completely consumed. The Grignard reagent (CypPrMgBr) was cooled to 0 °C by using an ice bath and compound 2 (5.0 g, 19.8 mmol, 1.0 eq.) dissolved in anhydrous THF (30 mL) was added. The resulting mixture was stirred at reflux overnight under N_2_-atmosphere. The mixture was then quenched with a saturated solution of NH_4_Cl (100 mL). Diethyl ether (100 mL) was added, and the mixture was stirred for 15 min at room temperature. The layers were separated, and the aqueous layer was extracted with diethyl ether (1× 25 mL). The organic layers were combined, washed with brine (50 mL), collected, dried with anhydrous Na_2_SO_4_, filtered, and concentrated in vacuum to give a yellow oil (6.4 g). The crude was used as such in the next step without purification.

After the Grignard reaction, the cyclopropyl ring was opened by using hydrochloric acid in acetic acid. Thus, the crude material was dissolved in glacial acetic acid (100 mL) and a 20% aqueous solution of HCl (100 mL) was added. The resulting solution was stirred for 2 h at room temperature. The acidic mixture was then concentrated in vacuum and the crude was dissolved in DCM (100 mL). The solution was washed with a saturated solution of NaHCO_3_ (3× 100 mL), water (100 mL), and brine (25 mL). The organic layer was collected, dried with anhydrous Na_2_SO_4_, filtered, and concentrated in vacuum to give 8-(benzyloxy)-4-(3-chloropropyl)-1,2-dihydronaphthalene and (E)-5-(benzyloxy)-1-(3-chloropropylidene)-1,2,3,4-tetrahydronaphthalene (3) as a brown oil (5.4 g, 87% of yield). No purification was performed regarding the presence of isomers and thus, the isolated material was used as such in the next step.

Step 3: Mixture of 2-(3-(5-(benzyloxy)-3,4-dihydronaphthalen-1-yl)propyl)-6,7-dimethoxy-1,2,3,4-tetrahydroisoquinoline and (*E*)-2-(3-(5-(benzyloxy)-3,4-dihydronaphthalen-1(2H)-ylidene)propyl)-6,7-dimethoxy-1,2,3,4-tetrahydroisoquinoline (4).

The crude mixture containing compound **3** (5.4 g, 19.8 mmol, 1.0 eq.) was dissolved in DMF (100 mL). Anhydrous Na_2_CO_3_ (6.3 g, 59.4 mmol, 3.0 eq.) was added along with commercially available (Combi Blocks) 6,7-dimethoxy-1,2,3,4-tetrahydroisoquinoline (5.75 g, 29.7 mmol, 1.5 eq.). The mixture was stirred overnight at 100 °C under N_2_-atmosphere. The mixture was allowed to cool down to room temperature. The mixture was partitioned between ethyl acetate (250 mL) and water (250 mL). The organic layer was collected, washed with water (3× 100 mL), collected, dried with anhydrous Na_2_SO_4_, filtered, and concentrated in vacuum to give (Z)-2-(3-(5-(benzyloxy)-3,4-dihydronaphthalen-1-yl)propyl)-6,7-dimethoxy-1,2,3,4-tetrahydroisoquinoline and (E)-2-(3-(5-(benzyloxy)-3,4-dihydronaphthalen-1(2H)-ylidene)propyl)-6,7-dimethoxy-1,2,3,4-tetrahydroisoquinoline mixture (4) compounds as a brown oil (8.2 g, quantitative). No purification was performed due to the presence of isomers and thus, the isolated material was used as such in the next step.

Step 4: 2-(3-(5-(benzyloxy)-1,2,3,4-tetrahydronaphthalen-1-yl)propyl)-6,7-dimethoxy-1,2,3,4-tetrahydroisoquinoline (5). Subsequently, a hydrogenation step was carried out to reduce double bonds of both isomers and to cleave the benzyl protecting group in one step. Therefore, the isomeric mixture 4 (8.2 g, 17.5 mmol, 1.0 eq.) was dissolved in a mixture of absolute ethanol (100 mL) and DMF (100 mL). This mixture was stirred under N_2_-atmosphere for 15 min. To this mixture was added, palladium on carbon (Pd/C) (10 wt% loading, 1.86 g, 1.75 mmol, 0.1 eq.). The resulting mixture was stirred for 48 h at room temperature under H_2_-atmosphere (atmospheric pressure) to remove the benzyl group. The mixture was filtered over a thin path of Celite and the filtrate was concentrated in vacuum, dissolved in EtOAc (100 mL), washed with water (4× 100 mL), and brine (100 mL). The organic layer was collected, dried with anhydrous Na_2_SO_4_, filtered, and concentrated in vacuum to give the crude containing the phenol. The crude was purified via silica column chromatography (DCM: MeOH, 95:5). The fractions that contain the pure compound were concentrated in vacuum to afford 5-(3-(6,7-dimethoxy-3,4-dihydroisoquinolin-2(1H)-yl)propyl)-5,6,7,8-tetrahydronaphthalen-1-ol (5) compound as an off-white foam (1.82 g, 25% yield calculated from benzylated intermediate (2)). ^1^H NMR (300 MHz, CDCl_3_) δ 6.98 (t, J = 7.8 Hz, 1H), 6.77 (d, J = 7.7 Hz, 1H), 6.62–6.49 (m, 3H), 3.84 (s, 3H), 3.83 (s, 3H), 3.57 (s, 2H), 2.88–2.45 (m, 9H), 1.92–1.57 (m, 8H).

Step 5: 2-(3-(5-(2-fluoroethoxy)-1,2,3,4-tetrahydronaphthalen-1-yl)propyl)-6,7-dimethoxy-1,2,3,4-tetrahydroisoquinoline (non-labelled MC225)

Compound 5 (50.0 mg, 1 eq, 131 μmol) was dissolved in DMF (3 mL) and sodium hydride (15.7 mg, 5 eq, 655 μmol) was added to the dissolved compound. The resulting mixture was stirred for 20 min until no further gas evolution was observed. 1-Bromo-2-fluoroethane (33.3 mg, 20 μL, 2 eq, 262 μmol) was added drop-wise to the reaction mixture. The reaction was monitored by LC-MS. After 20 min of stirring at room temperature, the reaction was complete. The reaction mixture was quenched by addition to a mixture of water/ ethylacetate (50 mL/50 mL). The organic layer was separated and the aqueous layer extracted with ethyl acetate (2 × 100 mL). The combined organic layers were dried over sodium sulfate and evaporated under reduced pressure. The crude material was purified by automated column chromatography yielding non-labelled MC225 (35.0 mg, 62.5%).^1^H NMR (299 MHz, CDCl_3_) δ 7.08 (t, *J* = 7.9 Hz, 1H), 6.84 (d, *J* = 7.8 Hz, 1H), 6.63 (d, *J* = 8.1 Hz, 1H), 6.59 (s, 1H), 6.52 (s, 1H), 4.90–4.77 (m, 1H), 4.75–4.57 (m, 1H), 4.24 (dd, *J* = 5.0, 3.4 Hz, 1H), 4.20–4.10 (m, 1H), 3.84 (s, 3H), 3.83 (s, 3H), 3.56 (s, 2H), 2.93–2.76 (m, 3H), 2.76–2.63 (m, 3H), 2.58–2.46 (m, 2H), 1.98–1.52 (m, 9H).

### Synthesis mesylate precursor (Fig. 3)

Step 1: 2-(3-(5-(2-(benzyloxy)ethoxy)-1,2,3,4-tetrahydronaphthalen-1-yl)propyl)-6,7-dimethoxy-1,2,3,4-tetrahydroisoquinoline (6).

**Fig. 3:**
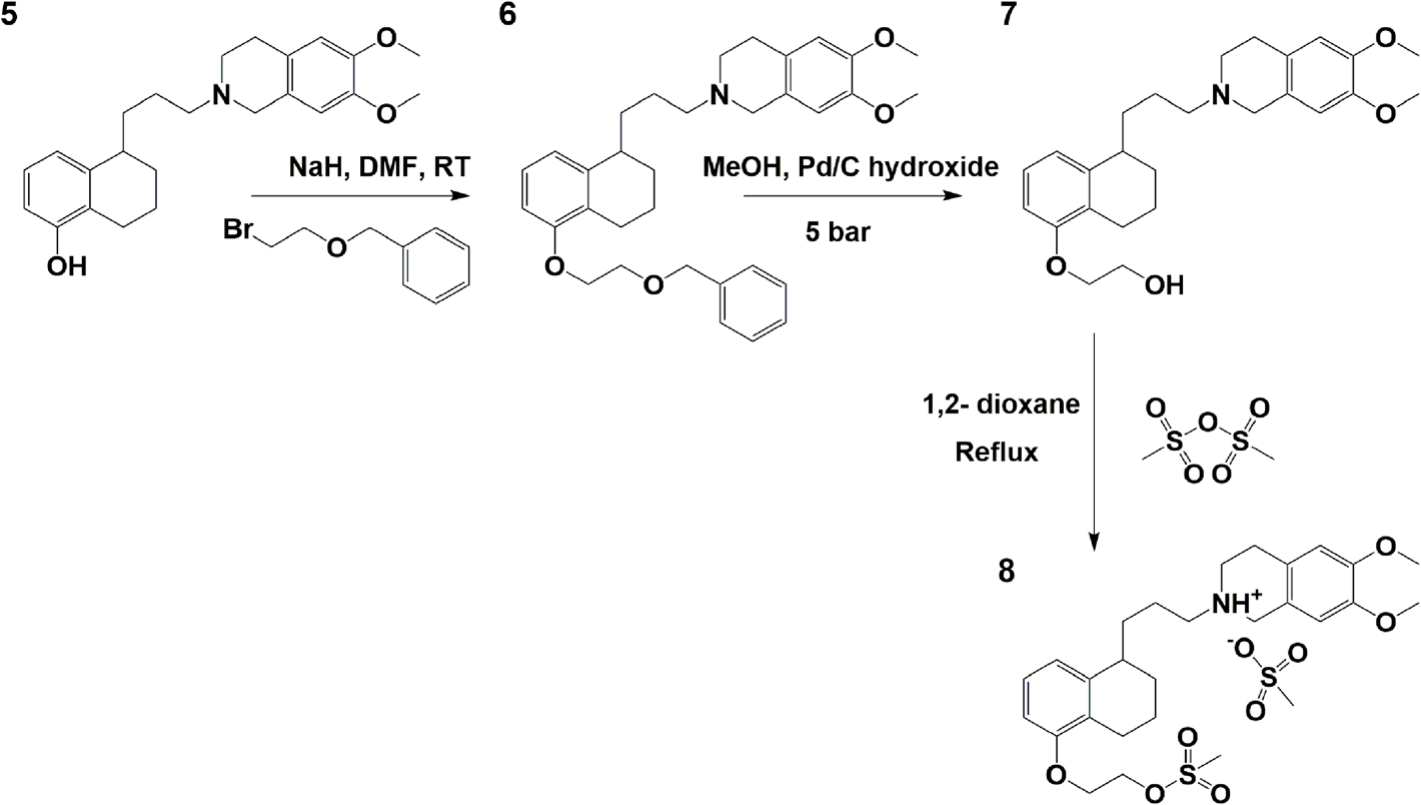
Synthesis of the MC225 mesylate precursor as its mesylate salt

5-(3-(6,7-Dimethoxy-3,4-dihydroisoquinolin-2(1H)-yl)propyl)-5,6,7,8-tetrahydronaphthalen-1-ol (**5**) (150.0 mg, 1 eq, 393.2 μmol) (phenol precursor or 5) was dissolved in DMF (12 mL) and sodium hydride (47.18 mg, 5 eq, 1.97 mmol) was added to the dissolved compound. The resulting mixture was stirred for 2 h until no gas evolution appeared anymore. ((2-Bromoethoxy)methyl) benzene (169 mg, 0.124 mL, 1.99 eq, 784 μmol) was added drop-wise to the reaction, and the progress of the reaction was monitored by LC-MS. After 20 min of stirring the reaction was completed according to LC-MS. The reaction mixture was quenched by adding to a mixture of water/ ethyl acetate (50 mL/50 mL). The organic layer was separated, and the aqueous layer was extracted with ethyl acetate (2 × 100 mL). The combined organic layers were dried over anhydrous Na_2_SO_4_ and evaporated under reduced pressure. The compound was analyzed by HPLC-MS. The crude material was purified by automated column chromatography (Biotage Purification System, Uppsala, Sweden) with a gradient of 0% to 50% ethyl acetate in heptane yielding 2-(3-(5-(2-(benzyloxy)ethoxy)-1,2,3,4-tetrahydronaphthalen-1-yl)propyl)-6,7-dimethoxy-1,2,3,4-tetrahydroisoquinoline (**6**) (165 mg, 84.4%). ^1^H NMR (300 MHz, CDCl_3_) δ 7.43–7.28 (m, 5H), 7.07 (t, *J* = 7.9 Hz, 1H), 6.81 (d, *J* = 7.7 Hz, 1H), 6.67–6.61 (m, 1H), 6.59 (s, 1H), 6.52 (s, 1H), 4.65 (s, 2H), 4.14 (dd, *J* = 5.8, 4.1 Hz, 2H), 3.90–3.78 (m, 8H), 3.55 (s, 2H), 2.91–2.76 (m, 3H), 2.76–2.62 (m, 4H), 2.58–2.44 (m, 2H), 1.91–1.60 (m, 8H).

Step 2: 2-((5-(3-(6,7-dimethoxy-3,4-dihydroisoquinolin-2(1H)-yl)propyl)-5,6,7,8-tetrahydronaphthalen-1-yl)oxy)ethan-1-ol (**7**).

A solution of **6** (150.00 mg, 1 eq., 290.87 μmol) in methanol (12 mL) and acetic acid (0.5 mL) was stirred under N_2_ for 20 min. Pd/C (20.00 mg, 0.5 Eq, 29.09 μmol) was added to the solution and the reaction was stirred overnight at 5 bar H_2_ pressure. After 3 days of stirring full conversion was observed. The mixture was filtered through a pad of Celite and the organic solvents were removed by rotary evaporation. The residue was dissolved in ethyl acetate and washed with a potassium bicarbonate solution. The organic layer was dried over anhydrous Na_2_SO_4_, filtered, and evaporated under reduced pressure yielding 2-((5-(3-(6,7-dimethoxy-3,4-dihydroisoquinolin-2(1H)-yl)propyl)-5,6,7,8-tetrahydronaphthalen-1-yl)oxy)ethan-1-ol (7) (100.20 mg, 81%). ^1^H NMR (299 MHz, cdcl_3_) δ 7.08 (t, *J* = 7.9 Hz, 1H), 6.84 (d, *J* = 7.8 Hz, 1H), 6.65 (d, *J* = 8.0 Hz, 1H), 6.59 (s, 1H), 6.52 (s, 1H), 4.07 (dd, *J* = 5.2, 3.7 Hz, 2H), 3.97 (dd, *J* = 5.2, 3.6 Hz, 2H), 3.84 (s, 3H), 3.83 (s, 3H), 3.56 (s, 2H), 2.89–2.76 (m, 3H), 2.76–2.65 (m, 3H), 2.57–2.46 (m, 2H), 1.93–1.51 (m, 9H).

Step 3: 6,7-dimethoxy-2-(3-(5-(2-((methylsulfonyl)oxy)ethoxy)-1,2,3,4-tetrahydronaphthalen-1-yl)propyl)-1,2,3,4-tetrahydroisoquinolin-2-ium methanesulfonate (8).

Compound **7** (105.00 mg, 1 eq., 246.73 μmol) was used without further purification and was dissolved in 1,4-dioxane (8 mL) and methanesulfonic anhydride (45.13 mg, 1.05 eq., 259.06 μmol) was added to the mixture. The resulting mixture was heated to reflux during 1 week until the reaction was complete. The reaction mixture was evaporated under reduced pressure, and 6,7-dimethoxy-2-(3-(5-(2-((methylsulfonyl)oxy)ethoxy)-1,2,3,4-tetrahydronaphthalen-1-yl)propyl)-1,2,3,4-tetrahydroisoquinolin-2-ium methanesulfonate (8) MC225 precursor was isolated as its mesylate salt. At this stage, we could not isolate intermediate 8 in pure form (93 mg, 75% pure,) and decided to use it as such for the radiolabeling. ^1^H NMR (299 MHz, CD_3_OD) δ 7.07 (q, *J* = 7.7 Hz, 1H), 6.81 (m, 3H), 6.71 (t, *J* = 7.3 Hz, 1H), 4.61–4.54 (m, 1H), 4.49 (d, *J* = 15.1 Hz, 1H), 4.33–4.16 (m, 3H), 4.01 (dd, *J* = 5.4, 4.3 Hz, 2H), 3.88 (dd, *J* = 5.5, 4.2 Hz, 2H), 3.82 (m, 6H), 3.80–3.70 (m, 2H), 3.31 (m, 10H), 2.01–1.65 (m, 8H).

### [^18^F]fluoride production

[^18^F]Fluoride was produced using a cyclotron (IBA Cyclone, Louvain-la-Neuve, Belgium) by the nuclear reaction ^18^O(p,n)^18^F.

### Automated synthesis (Fig. 4)

The synthesis was automated using two different modules the IBA Synthera module (Fig. 5) and the Modular Lab PharmTracer from Eckert & Ziegler (Fig. 6). Aqueous [^18^F]fluoride was trapped on Sep-Pak Light Accell Plus QMA anion exchange cartridge (Waters, Milford, USA) previously conditioned with 5 ml of Na_2_CO_3_ 1.4%, washed with 10 ml of H_2_O for injection, and then dried with a nitrogen stream. [^18^F]Fluoride was eluted using a solution of 1.3 ± 0.12 mg K_2_CO_3_ and 7.1 ± 0.26 mg Kryptofix 222 (K_2.2.2_) in 1 ml of ACN/H_2_O (8:2). Azeotropic drying of the [^18^F]KF/K_2.2.2_ complex was performed at 110 °C under an argon flow and adding 1 ml of ACN. After the evaporation of the solvent, the procedure was repeated twice using 0.5 ml of ACN.

**Fig. 4:**
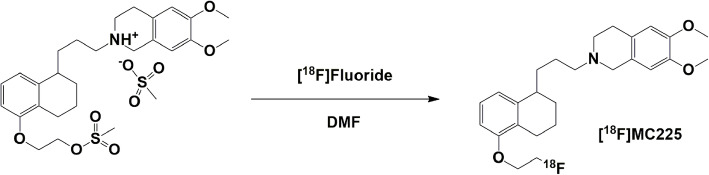
One-step radiosynthesis of [^18^F]MC225 using the MC225 mesylate precursor

**Fig. 5:**
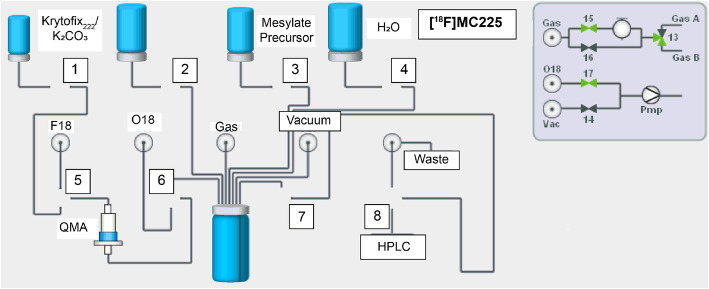
Scheme IBA Synthera module for the 1-step synthesis of [^18^F]MC225

**Fig. 6:**
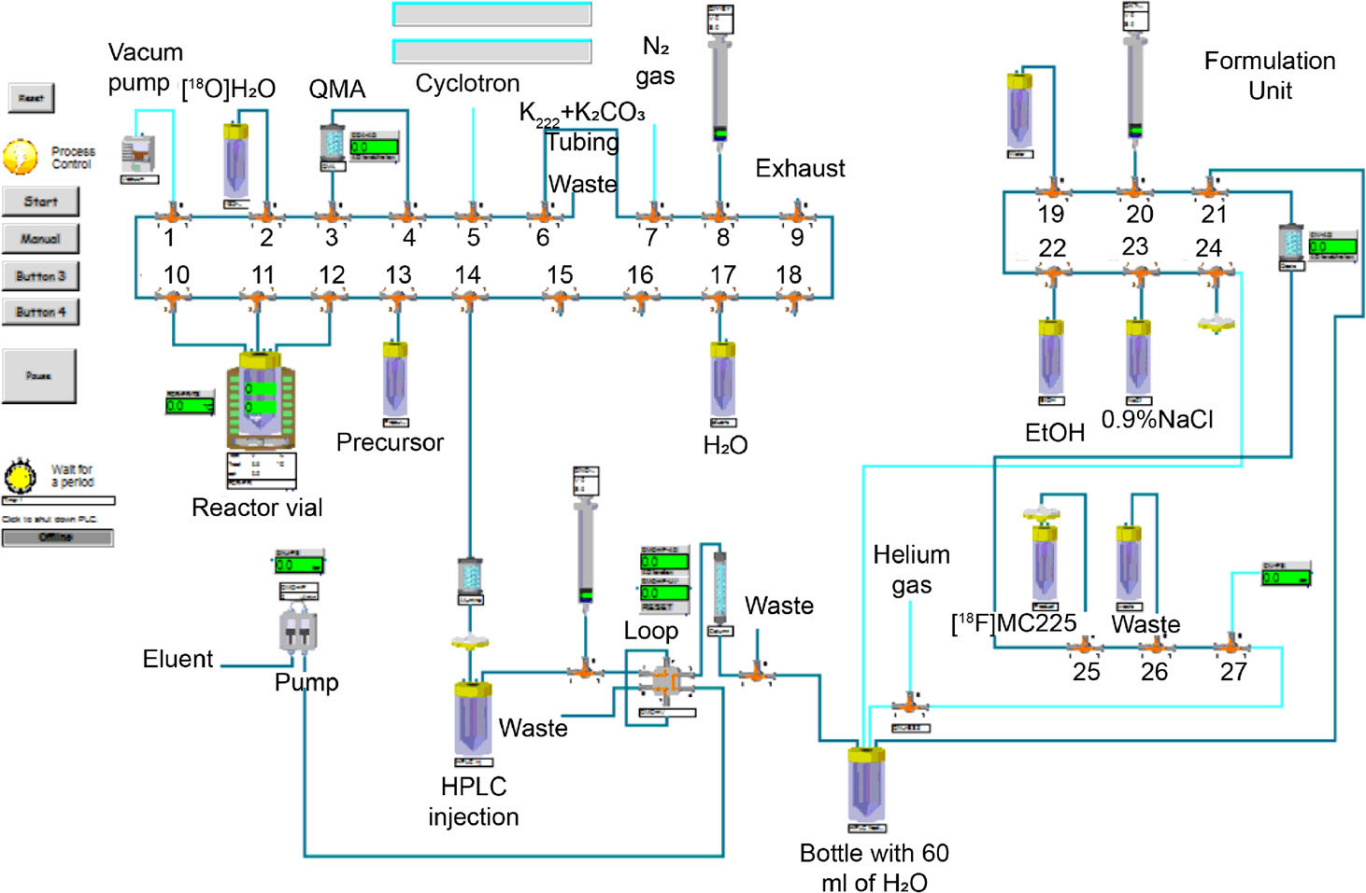
Scheme of Eckert & Ziegler module for the synthesis of [^18^F]MC225 using a 1-step procedure

Immediately after the azeotropic drying, 1 mg of mesylate precursor dissolved in 1 ml DMF (or 0.5 ml in the case of E&Z) was added to the reactor vial containing the dried [^18^F]KF/K_2.2.2_ complex. The reaction was heated at 140 °C for 30 min and after cooling down the solution 1 ml of H_2_O was added (1.3 ml in case of E&Z). The crude reaction was injected onto the semi-preparative HPLC and the product was collected after 10–11 min.

Purification of the crude reaction was done in a semi-preparative HPLC system using a Symmetryshield RP8 5 μm 7.8 × 300 mm column. 0.1 M NaOAc/ACN (5.5/4.5) (v/v) (pH = 4.7) were used as eluent at a flow of 3 ml/min. The UV signal was measured at a wavelength of 215 nm.

### Formulation of the product

Formulation of the product was done as previously described (Savolainen et al., 2015). Briefly, the desired product eluted at 10–11 min from the Prep-HPLC was collected in 60 ml of sterile H_2_O in an 80 ml bottle. The mixture was mixed with Helium and the mixture was passed through an Oasis HLB 1 cm^3^ (30 mg) extraction cartridge where the product was trapped. The cartridge was washed twice with 8 ml sterile H_2_O and the product was eluted with 1 ml of ethanol. Next, 4 ml of 0.9% NaCl was passed through the cartridge to formulate the final product. The solution was filtered through a Millipore Millex LG Filter (0.2 μm) before collection in a sterile vial.

### Quality control methods

Quality control was executed with a Waters Acquity H-class UPLC system (Milford, CT, USA) as previously described (Savolainen et al., 2017). The system used a Berthold FlowXStar LB 513 as a radioactivity detector (Bad Wildbad, Germany) and a Waters Acquity UPLC BEH Shield RP18 1.7 μm (3.0mmx50mm) column. The product eluted after 3.5 min using ACN / 10 mM NH_4_CO_3_ (pH = 9.5) (50/50) at a flow rate of 0.8 ml/min. The UV detection was set to 215 nm. The reference compound (non-labelled MC225) was used to prepare a calibration curve to know the amount of non-labelled compound and thus the molar activity (A_m_) of the final product at the end of the radiosynthesis.

## Results and discussion

This study aimed to develop a new synthesis method to produce the PET radiotracer [^18^F]MC225 via a 1-step synthesis. To this purpose, a mesylate precursor has been developed to facilitate direct ^18^F-fluorination yielding [^18^F]MC225.

This mesylate precursor (8) was synthesized from the phenol precursor (5) which was previously used in the 2-step synthesis of [^18^F]MC225 (Savolainen et al., 2015). Phenol precursor (5) was produced via an alternative synthesis which uses a benzyl-protected tetralone (2). The benzyl protecting group was chosen to reduce both the double bonds of both isomers (4) and to cleave the benzyl protecting group in one step. Palladium on carbon (Pd/C) was used as a catalyst and the hydrogenation was performed at atmospheric pressure. Full reduction of the double bonds was observed after stirring the mixture for 2 h at room temperature. The reaction was run for 48 h to remove the benzyl group. Moreover, the benzylated species were more stable than the unprotected phenol. At the end of the synthesis, the phenol precursor (5) was isolated and purified via column chromatography and obtained in a 25% yield (calculated from intermediate 2, Fig. 2).

The development of the mesylate precursor (8) was challenging. Firstly, the deprotection of the benzyl group of compound 6 was facing difficulties. Compound 6 seems to be unstable on silica thus reaction needs to achieve quantitative conversion to continue with the next step without further purification. The reaction was slow and different batches of Pd/C and Pd/C hydroxide were added to speed up the reaction and to obtain a maximum conversion. The reaction took more than 1 week to reach full conversion. Moreover, the reaction mixture needed to be slightly acidic to be able to reach completion, thus acetic acid was added. In the following step, compound **7** was used without further purification, and therefore, it was dissolved in dioxane and reacted with methanesulfonic anhydride at reflux. However, the mesylate precursor was not stable. Especially basic workup of the reaction mixture resulted in the elimination of the mesylate. Several strategies were explored to stabilize compound **8**. Eventually, it was observed that the mesylate salt formed during the reaction with methanesulfonic anhydride was reasonably stable and helped to stabilize the precursor for a longer time. However, the final mesylate precursor showed several impurities (purity 75%). Therefore, several attempts were made to improve purity e.g., via RP-automated column chromatography, prep HPLC and precipitation. All these attempts were unsuccessful leading to the deterioration of the product on the column or the isolation of impure product in very low yield after precipitation. Thus, the crude reaction mixture was evaporated and used as such for the labeling experiments.

**Fig. 7:**
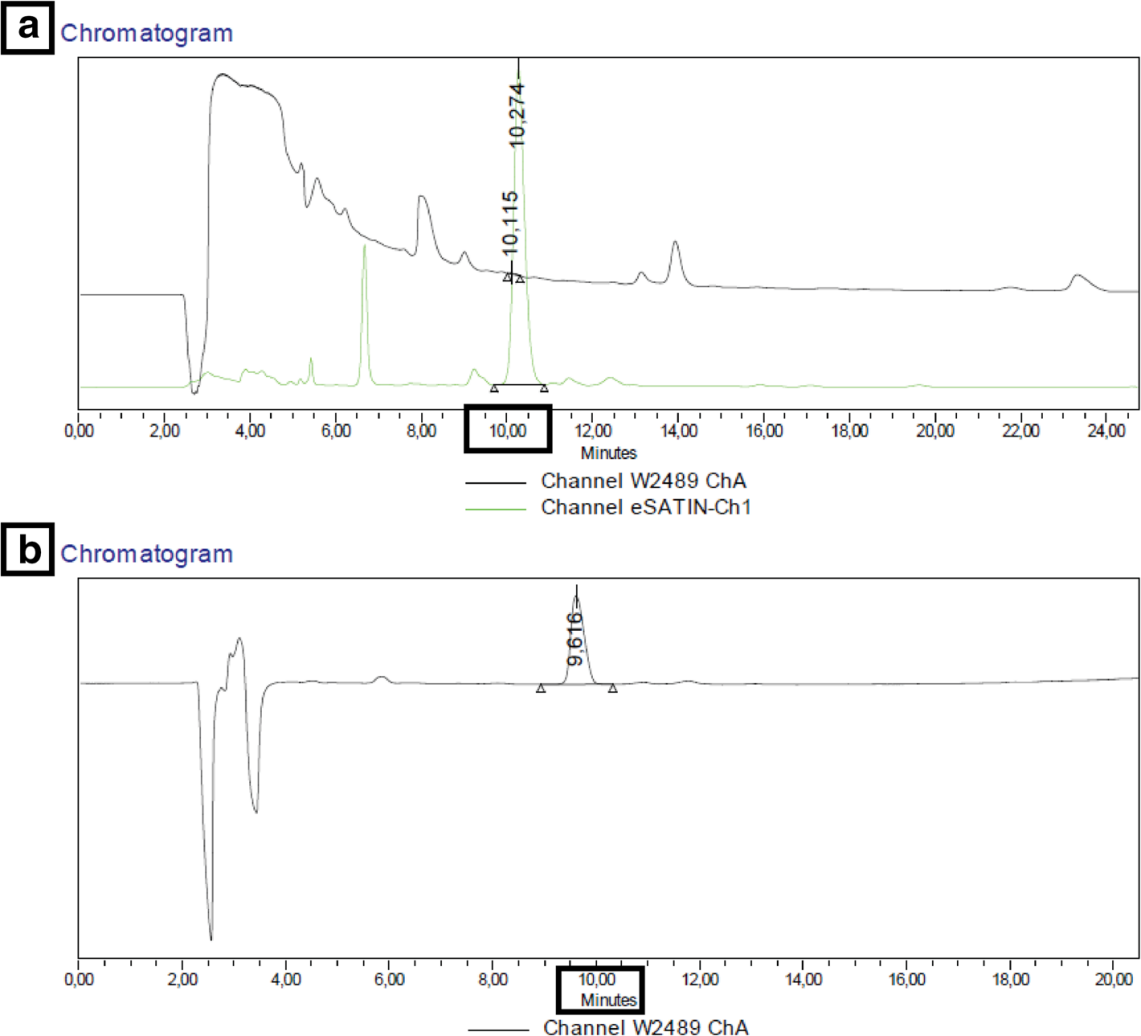
**a** Crude reaction (30 min at 140 °C), the product appeared after 10–11 min injection (the  green  signal represents the radioactivity and the black signal the UV) and **b** retention of reference compound MC225 (10–11 min) (black signal represents UV)

The radiolabeling was performed by adding the mesylate precursor to the dried [^18^F]KF/K_2.2.2_ complex and heating at 140 °C for 30 min. After the radiochemistry reaction, the crude mixture was purified using semi-preparative HPLC (as described in the methods) (Fig. 7).

The automated synthesis was performed using two modules: IBA Synthera Synthesis Module and the Modular Lab PharmTracer (Eckert & Ziegler). The best results were obtained with IBA Synthera module. The final product produced by both modules was analyzed by UPLC. Table 1 shows the results of both synthesis modules. The total synthesis time was 20 min shorter in the Synthera module than with E&Z module, mainly because of the faster drying process of the [^18^F]KF/K_2.2.2_ complex. The radiochemical purity of [^18^F]MC225 and the radiochemical yield (RCY) corrected for decay were higher in the IBA module. However, a high A_m_ of the final product was obtained with both modules. Regarding the preparation for the synthesis, the set-up of the synthesis was easier and quicker in the IBA Synthera than in the E&Z module. Although the Modular Lab PharmTracer from E&Z allows the configuration of multi-step and complicated synthesis by the combination of various disposable components, the amount of tubing and valves make the preparation more laborious. In the case of Synthera, the cartridge provides the connections and only the reagents must be placed in the right position during the preparation, thus the time spent in the preparation is shorter. For this reason, Synthera may be a preferable module to perform this synthesis. Independently of the module, we could produce a dose of 407 ± 263 MBq of [^18^F]MC225 in a single run starting from 15 to 20 GBq of [^18^F]fluoride.

**Table 1:** Characteristics of the 1-step synthesis performed in Eckert & Ziegler and IBA Synthera. Values reported in mean ± SD

Procedure	Total Reaction time (min)	Drying time (min)	RCY (%)	% Area[^18^F]MC225 (UPLC)	Molar Activity (A_m_) (GBq/ mmol)
Eckert & Ziegler (*n* = 9)	120.44 ± 26.33	20.33 ± 5.89	3.76 ± 2	91.70 ± 3.8	> 100,000
IBA synthera (*n* = 9)	106.09 ± 13.06	10.44 ± 0.73	6.53 ± 3	97.14 ± 1.12	> 100,000

**Fig. 8:**
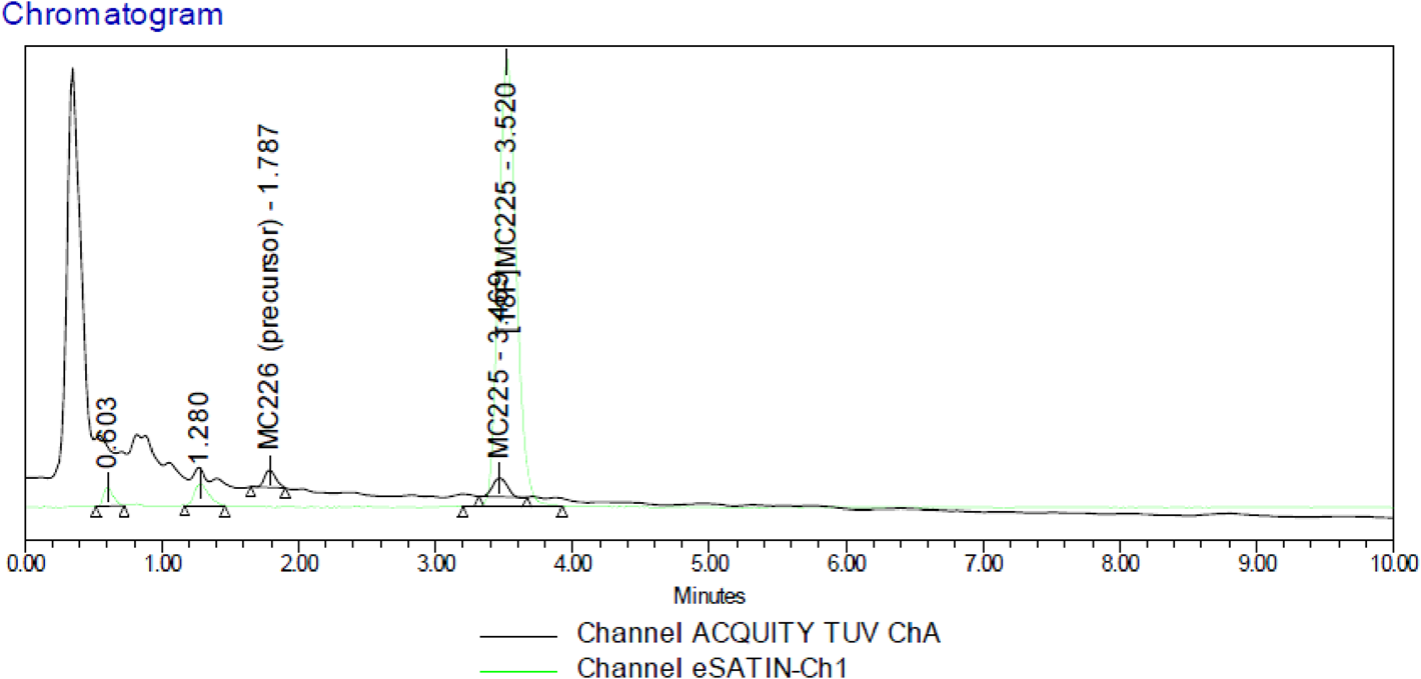
Final product injected into the UPLC system. [^18^F]MC225 elutes at 3.5 min

The final product collected from the semi-preparative HPLC was injected into the UPLC system to perform quality control. Figure 8 shows an example of UPLC chromatography that shows the desired product appearing at 3.5 min after the injection.

This study provides an alternative method for the production of [^18^F]MC225 using a 1-step approach. However, to obtain a GMP compliant synthesis a suitable purification of the mesylate precursor is still warranted. Nevertheless, an automated synthesis using the mesylate precursor and the IBA Synthera module produced [^18^F]MC225 in a reliable and simple manner. Quality control performed with UPLC ensures an adequate purity and A_m_ of the final product.

The main benefit of the 1-step synthesis is that it is a more simple and easier procedure compared to the two-step synthesis and it allows straightforward automation in modules similar to the IBA Synthera. The yields, purity, molar activity, and reaction time obtained were similar with both methods. The 1-step synthesis may be a more attractive method for the translation of the synthesis to other PET centers.

## Conclusion

Overall, the production of [^18^F]MC225 by the 1-step synthesis using the mesylate precursor and the IBA Synthera module seems to be the most successful automated method. The highest radiochemical yield and the adequate purity and A_m_ of the final product will enable the use of this procedure for GMP productions.

## Data Availability

The datasets used and/or analyzed during the current study are available from the corresponding author on reasonable request.

## Abbreviations

ACN: Acetonitrile
NH_4_Cl: Ammonium chloride
BBB: Blood-brain barrier
CNS: Central Nervous System
CypPrMgBr: Cyclopropyl magnesium bromide
DCM: Dichloromethane
DMF: Dimethylformamide
EtOAc: Ethyl acetate
GMP: Good Manufacturing Practice
HPLC-MS: High-performance liquid chromatography-mass spectroscopy
h: Hour
HCl: Hydrochloric acid
H_2_-atmosphere: Hydrogen atmosphere
LC-MS: Liquid chromatography-mass spectroscopy
MeOH: Methanol
μm: Micrometer
mg: Milligram
ml: Milliliter
min: Minute
A_m_: Molar activity
nm: Nanometer
N_2_-atmosphere: Nitrogen atmosphere
Pd/C: Palladium on carbon
P-gp: P-glycoprotein
PET: Positron Emission Tomography
K_2_CO_3_: Potassium carbonate
H-NMR: Proton nuclear magnetic resonance
RCY: Radiochemical yield
RP-automated column chromatography: Reverse Phase automated column chromatography
NaOAc: Sodium acetate
NaHCO_3_: Sodium bicarbonate
Na_2_CO_3_: Sodium carbonate
NaCl: Sodium chloride
Na_2_SO_4_: Sodium sulfate
THF: Tetrahydrofuran
TLC: Thin Layer Chromatography
UPLC: Ultra-performance liquid chromatography
UV: Ultraviolet
H_2_O: Water
